# Factors associated with Motorcyclist risky riding behaviors in southern Iran

**Published:** 2019-08

**Authors:** Seyed Taghi Heydari, Yaser Sarikhani, Arash Mani, Leila Mohammadi, Mehrdad Vossoughi, Kamran Bagheri Lankarani

**Affiliations:** ^*a*^Health Policy Research Center, School of Medicine, Shiraz University of Medical Sciences, Shiraz, Iran.; ^*b*^Student Research Committee, Shiraz University of Medical Sciences, Shiraz, Iran.; ^*c*^Research Center for Psychiatry and Behavior Sciences and Community Based Psychiatric Care Research Center, Shiraz University of Medical Sciences, Shiraz, Iran.; ^*d*^Department of Dental Public Health, Oral and Dental Disease Research Center, School of Dentistry, Shiraz University of Medical Sciences, Shiraz, Iran.

## Abstract

**Background:**

Motorcyclist’s behavior has significant relationship with increased accidents mortality rate among them. Determining the risky behaviors of motorcycle drivers is essential for preventing riders and other community members from accident risks. The aim of this study was to determine the risky driving behaviors of motorcyclists in Fars province of Iran in 2017.

**Methods:**

In this cross sectional study was done in 2017 total number of 1195 motorcyclist from Fars province were included. In this study we collected the data using the Persian version of Motorcycle riding behavior questionnaire (MRBQ) was used to study the risky behaviors of motorcycle riders while riding and a checklist designed by the researchers for demographic variables. SPSS 22 was used to analyze the data.

**Results:**

All participants were male and the mean age was 28.28±8.56 years. The mean score of MRBQ was 53.54±23.44. The mean score of MRBQ was higher among the motorcyclists with the history of accident in the past year, those who had more pleasure while driving motorcycle, those who used alcohol or substance two hours before driving, those who used more powerful motorcycle, drivers with lower education level, and those with higher usage of motorcycle (P<0.001).

**Conclusions:**

Finding of this study indicated that motorcyclists with the higher score of MRBQ are at the higher risk of traffic accidents. Therefore, designing and implementing appropriate training programs before giving driving license could improve knowledge and attitude of drivers toward the risky driving behavior.

**Keywords:**

Motorcycle riders, Risky behavior, Fars

